# Botulinum toxin treatment for multiple sclerosis and post-stroke spasticity in clinical practice: differences in injection doses and patterns

**DOI:** 10.3389/fresc.2026.1838788

**Published:** 2026-06-09

**Authors:** Paolo Manganotti, Alessandro Dinoto, Giulia Mazzon, Anna Favero, Lucrezia Rossi, Marta Cheli, Federica Palacino, Alessio Bratina, Antonio Bosco, Arianna Sartori

**Affiliations:** 1 Neurology Unit, Department of Medical, Surgical and Health Sciences, University of Trieste, Trieste, Italy; 2 Neurology Unit, Hospital Care Department of Medicine, Azienda Sanitaria Universitaria Giuliano Isontina (ASUGI), Trieste, Italy

**Keywords:** botulinum toxin (BoNT), multiple sclerosis, post-stroke spasticity, spasticity, symptomatic treatment

## Abstract

**Introduction:**

Botulinum Toxin Type A (BoNT-A) is a safe and effective treatment for spasticity in both multiple sclerosis (MS) and post-stroke spasticity (PSS). This single-center retrospective study compared injection patterns and dosages between these two conditions.

**Methods:**

Thirty-three people with MS (PwMS) and 55 people with PSS treated with BoNT-A at our outpatient spasticity clinic were analyzed. Clinical and demographic data, BoNT-A dosages, upper- and lower-limb dosing, patterns of injected muscles, and their respective dosages were collected. BoNT-A treatment doses and injection patterns were compared between groups. Patient-reported benefit was also recorded.

**Results:**

PwMS more frequently received treatment in lower limbs and lower total doses of BoNT-A. Patients with PSS were more commonly treated in distal upper-limb muscles. In the lower limbs, PwMS were more frequently treated in the adductor muscles and rectus femoris, while patients with PSS were more often treated in tibialis posterior. In MS, higher Expanded Disability Status Scale (EDSS) scores were associated higher total BoNT-A dosage.

**Discussion:**

MS-related spasticity mainly affects the lower limbs and less frequently upper limbs, and requires lower BoNT-A dosages compared with PSS. In contrast, PSS is characterized by more prominent distal spasticity, particularly in the upper limbs. Treatment patterns and BoNT-A dosing are influenced by these differences in spasticity distribution. We hypothesize that the need to preserve residual motor function, particularly in lower limbs, may have contributed to lower BoNT-A doses in MS, although not systematically investigated. Despite being only marginally assessed, treatment efficacy appeared to be similar in both groups.

## 1 Introduction

Spasticity is a frequently underestimated and undertreated complication of several neurological conditions, including stroke, multiple sclerosis (MS), and cerebral palsy, with a significant impact on patients' quality of life (1).

Its pathogenesis is highly complex and involves an imbalance between inhibitory and excitatory pathways (2), largely determined by the location of the underlying lesion (supraspinal, spinal, or mixed). Supraspinal mechanisms predominate in post-stroke spasticity (PSS), whereas they play a more limited role in MS, in which spasticity is mainly driven by a spinal involvement (3). These pathophysiological differences could reflect the distinct clinical features observed in PSS and MS-related spasticity.

PSS is a highly disabling condition with a significant impact on patients' quality of life (4). It usually develops 1–6 weeks after the cerebrovascular event, with greater severity within 1–3 months (5). Prevalence estimates of PSS vary across studies, as reported in a review by Wissel and colleagues: while early PSS (1–4 weeks post-stroke) affects 4%–27% of patients, this proportion rises to 17%–42.6% in the chronic phase (>3 months post-stroke) (6). Several risk factors associated with PSS development have been identified, including greater global disability (assessed by the modified Rankin Scale and Barthel Index), more severe paresis in the acute phase, sensory disturbances, and pain (7). According to the American Heart Association/American Stroke Association guideline for poststroke rehabilitation, PSS treatment includes stretching, splinting, oral medications, chemodenervation and botulinum toxin for focal spasticity (8). In particular, the latter is recommended as a first-line reversible treatment for focal PSS by several international guidelines (9, 10). Prompt identification of spasticity and its predictors is recommended to improve treatment outcomes (11).

In MS, spasticity represents a major clinical issue, as it may develop in 60%–84% of patients during the disease course (12). The clinical presentation of MS-related spasticity can be very heterogeneous and therefore requires tailored interventions (13). It is also often under-recognized and undertreated (14, 15). According to a 2016 international consensus (16), beyond physical therapy and the avoidance of aggravating factors, generalized MS-related spasticity should be treated with first-line oral antispastic therapies (e.g., baclofen, gabapentin, and tizanidine). Diazepam and dantrolene are considered second-line options (17) while cannabis-based oromucosal spray nabiximols is recommended as an add-on treatment when standard therapies are ineffective (18).

For focal spasticity, particularly in the lower limbs, the use of Botulinum Toxin Type A (BoNT-A) injections is supported by consistent evidence in the literature (19–25), while phenol injections represent a viable alternative (16).

BoNT-A acts as a muscle relaxant by blocking the presynaptic release of acetylcholine at the neuromuscular junction.

While BoNT-A is well recognized both for upper- and lower-limb spasticity, with class I, level A of evidence (8), studies in MS are less numerous, resulting in a lower level of evidence. Moreover, MS-related spasticity more commonly affects the lower limbs, and its treatment usually requires a careful balance between spasticity reduction and preservation of residual motor function, often requiring lower BoNT-A doses.

Nevertheless, data on BoNT-A dosing across different neurological conditions remain scarce. To date, only a few studies have compared patients with different conditions (MS, PSS, and cerebral palsy or traumatic brain injury and spinal cord injury) in terms of injected muscles and BoNT-A dosage (26–28).

To investigate differences in the management of BoNT-A between the two most prevalent conditions treated at our outpatient spasticity clinic, we conducted a single-center retrospective study. The primary aim was to compare clinical characteristics and treatment-related variables (injection patterns, treated muscle groups, and BoNT-A dosing strategies) between patients with MS-related spasticity and those with PSS. As a secondary aim, we also compared these variables between patients with ischemic and hemorrhagic stroke. Finally, exploratory analyses were performed to investigate variables associated with BoNT-A dosing.

## 2 Materials and methods

This study was conducted at the Spasticity Outpatient Clinic of the Neurology Unit, Cattinara Hospital, Italy from July 2015 to July 2019, in a chronic care setting. People with MS or PSS treated with BoNT-A were consecutively included. Patient referral was made by neurologists from the Post-Stroke Outpatient Clinic and the MS Clinic of our Neurology Unit.

Inclusion criteria were age ≥18 years and the absence of changes in any concomitant anti-spasticity treatments during the study period. Patients who did not provide informed consent and those with insufficient available information were excluded from the study. The study was approved by the local ethics committee and conducted in accordance with the principles of the Declaration of Helsinki.

### 2.1 BoNT-A treatment

Patients were treated with onabotulinumtoxinA (ONA, Botox®) or incobotulinumtoxinA (INCO, Xeomin®). BoNT-A dosage was expressed in units (U), and dilution was set at 1:2. Although a 1:1 conversion ratio between BoNT-A formulations has been previously suggested (29), more recent evidence indicates that no fixed doses equivalence exists between incobotulinumtoxinA and onabotulinumtoxinA; therefore, dosing should be individualized based on clinical goals and manufacturer recommendation (30, 31). Accordingly, analyses were performed separately for each product, while the 1:1 conversion ratio was applied exclusively to enable comparison across muscle groups and for correlation analysis with disability measures. All BoNT-A injections were performed by two experienced physicians (a neurologist and a neurologist/physiatrist), using ultrasound guidance and, in selected cases, electromyographic guidance. No pre-specified injection protocols were used; the selection of muscles and the amount of toxin injected were determined on a case-by-case basis, according to therapeutic goals (in particular, maintenance of residual function), physician-assessed severity of spasticity, and patients' perceived spasticity.

### 2.2 Clinical variables

Demographic and clinical data were collected, including age, sex, treatment duration, number of treatments, and disease duration. Disease severity was assessed using the National Institutes of Health Stroke Scale (NIHSS) for stroke patients and the Expanded Disability Status Scale (EDSS) for PwMS. For MS, disease form was collected according to classical classification (relapsing-remitting, secondary progressive, or primary progressive). Stroke etiology (ischemic or hemorrhagic) was also recorded. Type of spasticity was collected and classified as: upper limb monolateral spasticity, upper limb bilateral spasticity, lower limb monolateral spasticity, lower limb bilateral spasticity (paraspasticity), hemispasticity, and tetraspasticity.

The following variables for BoNT-A treatment were collected: total incobotulinumtoxinA and onabotulinumtoxinA dose, BoNT-A dose per injected muscle, number of treated limbs, number of treated limbs relative to number of limbs affected, and number of treated muscles. Upper- and lower-limb data were also collected separately: in particular, we collected incobotulinumtoxinA and onabotulinumtoxinA dose and treated spasticity distribution patterns (bilateral upper and/or lower limbs or ipsilateral upper and lower limbs). Due to the retrospective nature of the study, standardized scales for evaluation of BoNT-A efficacy were not available. Nevertheless, to provide an indication of treatment efficacy, we extracted patients’ self-reported benefits during follow-up visits through a review of medical records.

### 2.3 Statistical analysis

Continuous variables were reported as mean and standard deviation or median and range, according to their distribution. The Shapiro–Wilk test was used to assess normality. Clinical and treatment-related variables were compared between MS-related spasticity and PSS groups. Pearson's chi-square test or Fisher's Exact Test were used to compare categorical variables, as appropriate. The *T*-test or Mann–Whitney *U* test were used to compare continuous variables, according to their distribution, as appropriate. A similar sub-analysis was performed in stroke patients to compare the same variables between ischemic and hemorrhagic stroke.

Multivariate analyses were applied to evaluate baseline clinical variables that could be associated with BoNT-A treatment dose. In particular, linear regression analyses, with BoNT-A dosage (incobotulinumtoxinA and onabotulinumtoxinA) as the dependent variable, were performed. To also include disability scores as factors associated with total BoNT-A dosage, separate logistic regression analyses were repeated for PwMS and PSS groups.

Muscle-specific variables (percentage of treated muscles and BoNT-A dosage) were compared between MS-related spasticity and PSS groups using the aforementioned tests, and a Benjamini-Hochberg procedure was applied to control the false discovery rate (FDR).

Finally, we compared the clinical characteristics of subjects who reported a benefit from treatment with those who did not report a positive effect following BoNT-A injections. A logistic regression analysis was performed to identify clinical variables significantly associated with reported treatment efficacy.

Statistical significance was set at a *p*-value <0.05. All statistical analyses were performed using IBM SPSS Statistics version 31.0.

## 3 Results

### 3.1 Patients' characteristics and spasticity pattern

Data from 33 PwMS and 55 patients with PSS were collected. No differences were observed between groups in sex distribution, treatment duration, or number of injection cycles. PwMS were younger and had a longer disease duration compared with patients with PSS (Table 1). Regarding baseline spasticity patterns, PwMS presented a higher proportion of paraspasticity and tetraspasticity, while PSS patients showed a significantly higher proportion of monolateral upper limb spasticity and hemispasticity (Table 1). The number of limbs affected by spasticity was higher in PwMS group; moreover, PwMS were more frequently affected by lower limb spasticity compared with the PSS group, whereas the latter showed a higher proportion of patients with at least one upper limb affected (Table 1). The PSS group had a moderate disability, with a median NIHSS of 7, while PwMS had severe disability, with a median EDSS of 7. More than 75% of PwMS had a progressive disease form, and approximately 70% were not treated with DMDs (Table 1).

**Table 1:** Baseline demographic and clinical data, expressed as “median (range)” or “number (percentage)”.

Baseline patient characteristics	MS group (*n* = 33)	PSS group (*n* = 55)	*p*
Age (years)	53 (24–72)	70 (39–86)	<0.001^a^
Sex (m)	19 (57.6%)	41 (74.5%)	0.079^b^
Disease duration (years)	16 (2–48)	3 (0–29)	<0.001^a^
Treatment duration (years)	0 (0–3)	0 (0–4)	0.053^a^
Number of treatments	2 (1–12)	3 (1–11)	0.232^a^
Type of spasticity			<0.001^b^
Upper limb, monolateral	1 (3%)	23 (41.8%)	<0.001^b^
Upper limb, bilateral	1 (3%)	0 (0%)	0.375^c^
Lower limb, monolateral	7 (21.2%)	12 (21.8%)	1.000^b^
Lower limb, bilateral (paraspasticity)	14 (42.4%)	0 (0%)	<0.001^b^
Hemispasticity	1 (3%)	20 (36.4%)	<0.001^b^
Tetraspasticity	9 (27.3%)	0 (0%)	<0.001^c^
Upper limb spasticity (mono or bilateral)	12 (36.4%)	43 (78.2%)	<0.001^b^
Lower limb spasticity (mono or bilateral)	31 (93.9%)	32 (58.2%)	<0.001^b^
Total number of limbs affected by spasticity	2 (1–4)	2 (1–2)	<0.001^a^
NIHSS		7 (3–14)	
EDSS	7 (2–8.5)		
MS disease form			
Relapsing-remitting	8 (24.2%)		
Secondary progressive	20 (60.6%)		
Primary progressive	5 (15.2%)		
MS disease-modifying drugs			
No treatment	23 (69.6%)		
Glatiramer acetate	2 (6.1%)		
Dimethyl fumarate	2 (6.1%)		
Teriflunomide	2 (6.1%)		
Ocrelizumab	2 (6.1%)		
Azathioprine	1 (3%)		
Siponimod	1 (3%)		

MS, multiple sclerosis; PSS, post-stroke spasticity; NIHSS, National Institutes of Health Stroke Scale; EDSS, Expanded Disability Status Scale.

a Mann–Whitney *U* test.

b Pearson's chi-square.

c Fisher's exact test.

No differences were found between patients with ischemic (*n* = 44) and hemorrhagic stroke (*n* = 11) with respect to demographic characteristics, clinical data, or BoNT-A treatment variables (Supplementary Table S1).

### 3.2 Treated limbs and BoNT-A dose

PwMS were more frequently treated with incobotulinumtoxinA, had a greater number of treated limbs, but fewer injected muscles. Patients with PSS were more frequently treated in the upper limbs, whereas PwMS were more often treated in the lower limbs (Table 2). The discrepancy between limbs affected by spasticity and limbs treated with BoNT-A was higher in PwMS group, resulting in a lower proportion of patients treated in all affected limbs compared with PSS (57.6% vs. 98.2%) (Table 2).

**Table 2:** Botulinum toxin-A treated limbs, dose, and reported efficacy. Data are expressed as median “range” or “number (percentage)”.

Botulinum toxin-A treatment characteristics	MS group (*n* = 33)	PSS group (*n* = 55)	*p*
Toxin injected			
incobotulinumtoxina	24 (72.7%)	16 (29.1%)	<0.001^b^
onabotulinumtoxina	9 (27.3%)	39 (70.9%)	
Number of limbs injected	2 (1–2)	1 (1–2)	0.045^a^
Delta affected-treated limbs	0 (0–3)	0 (0–1)	<0.001^a^
Subjects treated in all spasticity affected limbs	19 (57.6%)	54 (98.2%)	<0.001^b^
Number of muscles injected	2 (1–3)	4 (1–8)	<0.001^a^
Patients treated in upper limb(s)	5 (15.2%)	43 (78.2%)	<0.001^b^
bilateral injections	2 (6.1%)	0 (0%)	0.138^c^
number of muscles injected	0 (0–3)	2 (0–7)	<0.001^a^
Patients treated in lower limb(s)	28 (84.8%)	30 (54.5%)	0.004^b^
Bilateral injections	16 (48.5%)	0 (0%)	<0.001^b^
Number of muscles injected	1 (0–3)	1 (0–4)	0.354^a^
Ipsilateral upper and lower limb injections	0 (0%)	18 (32.7%)	<0.001^b^
BoNT-A total dose (U)			
incobotulinumtoxina	100 (20–300)	230 (130–400)	0.001^a^
onabotulinumtoxinA	125 (50–200)	200 (25–400)	0.006^a^
Upper limbs max dose (U)			
incobotulinumtoxinA	*N* = 2, 195 (190–200)	*N* = 14, 175 (50–400)	0.747^a^
onabotulinumtoxinA	*N* = 3, 100 (70–200)	*N* = 29, 200 (50–400)	0.151^a^
Lower limb max dose (U)			
incobotulinumtoxinA	*N* = 22, 100 (20–250)	*N* = 9, 145 (50–200)	0.097^a^
onabotulinumtoxinA	*N* = 6, 75 (50–200)	*N* = 21, 150 (25–300)	0.131^a^
Patients reporting benefit from BoNT-A treatment	29 (90.6%)	45 (86.5%)	0.735^c^

MS, multiple sclerosis; PSS, post-stroke spasticity; BoNT-A, Botulinum toxin-A.

a Mann–Whitney *U* test.

b Pearson's chi-square.

c Fisher's exact test.

PwMS received significantly lower cumulative doses of both incobotulinumtoxinA (median: 100, range: 20–300 vs. 230, range: 130–400; *p* = 0.001), and onabotulinumtoxinA, (median: 125, range: 50–200 vs. 200, range: 25–400; *p* = 0.006).

No differences were observed in onabotulinumtoxinA and incobotulinumtoxinA doses when upper and lower limbs were analyzed separately (Table 2).

For incobotulinumtoxinA, regression analysis indicated that one independent variable explained 27% of the variance (*R*^2^ = 0.270 [*F*(1,38) = 14.08, *p* < 0.001]). PSS significantly predicted higher incobotulinumtoxinA dosage (*B* = 107.55, 95% CI: 49.53–165.56, *p* < 0.001). Sex, age, disease duration, and number of affected limbs were excluded from the model.

For onabotulinumtoxinA, results were similar: the regression indicated that PSS explained 14.4% of the variance (*R*^2^ = 0.144 [*F*(1,46) = 7.76, *p* = 0.008]). PSS significantly predicted higher onabotulinumtoxinA dosage (*B* = 92.55, 95% CI: 25.69–159.42, *p* = 0.008). Again, sex, age, disease duration, and number of affected limbs were excluded from the model.

To include disease severity assessed by EDSS and NIHSS, analyses were repeated separately for MS and PSS groups, with total BoNT-A dose (using a 1:1 conversion ratio between incobotulinumtoxinA and onabotulinumtoxinA, despite this not being universally accepted) as the dependent variable.

For total BoNT-A dosage in PwMS, regression analysis indicated that one independent variable explained 12.6% of the variance (*R*^2^ = 0.126 [*F*(1,31) = 4.47, *p* = 0.043]). In particular, EDSS (*B* = 18.98, 95% CI: 0.67–37.30, *p* = 0.043) significantly predicted higher BoNT-A dosage (Supplementary Figure S1). Sex, age, disease duration, and treatment duration were excluded from the model.

For total BoNT-A dosage in PSS, the overall model was not significant (*R*^2^ = 0.159 [*F*(4,50) = 2.36, *p* = 0.066]).

### 3.3 Treated muscles

**Figure 1:**
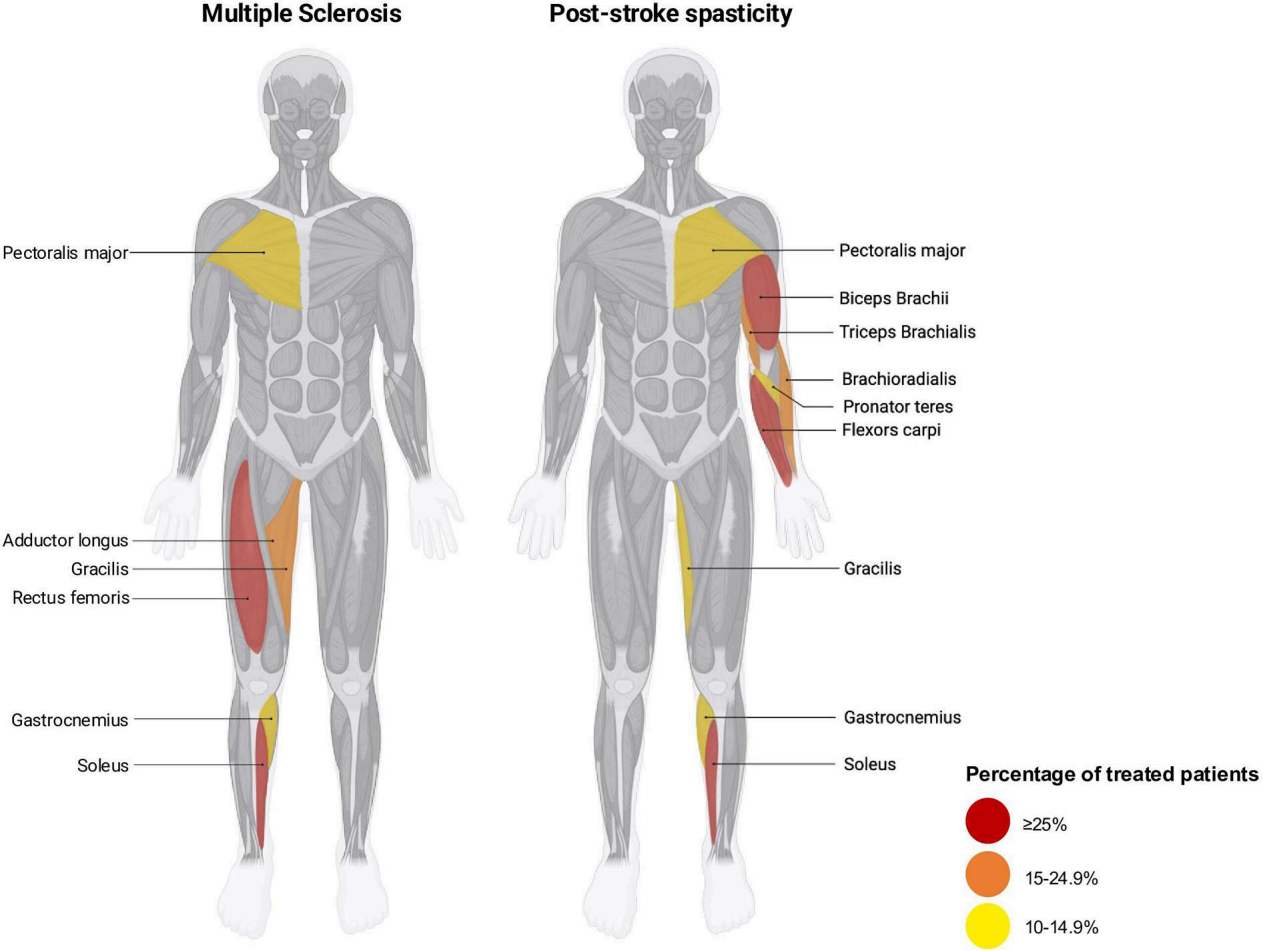
Representation of most commonly injected sites in multiple sclerosis and post-stroke spasticity. This Figure was created in BioRender. Sartori (2026) https://BioRender.com/vuizxo3.

**Figure 2:**
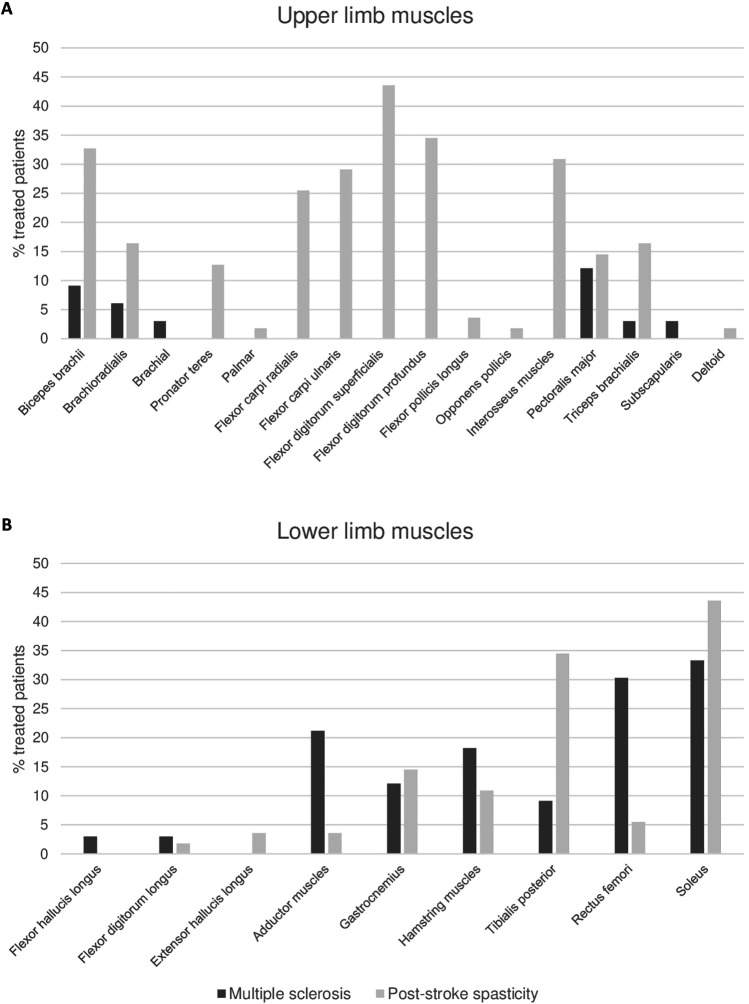
BoNT-A injected muscles in upper limbs (panel **A**) and lower limbs (panel **B**). Percentage of patients treated for each muscle in multiple sclerosis group and post-stroke spasticity group.

Regarding injection patterns and muscle-specific BoNT-A doses, PwMS were usually injected in proximal upper-limb muscles (pectoralis major, biceps brachii, and brachioradialis), while finger and wrist flexor muscles were not treated. Patients with PSS were most frequently injected in hand and wrist flexor muscles. In particular, the most commonly treated muscles were flexor digitorum superficialis and profundus, biceps brachii, interosseus muscles, flexor carpi ulnaris and radialis, and pronator teres (Figures 1, 2 panel A, Supplementary Table S2).

In the lower limbs, PwMS were more frequently injected in the adductor muscles and rectus femoris. Conversely, patients with PSS were more frequently injected in the tibialis posterior. Soleus muscle was treated in both groups but PSS required higher BoNT-A doses (Figures 1, 2 panel B, and Supplementary Table S2).

After application of the Benjamini–Hochberg procedure to control the FDR, flexor carpi radialis, flexor carpi ulnaris, flexor digitorum superficialis, flexor digitorum profundus, interosseous muscles, and tibialis posterior remained significantly more frequently treated in the PSS group, while rectus femoris was more frequently injected in PwMS.

### 3.4 Treatment efficacy

Efficacy assessment was limited, as it was based solely on patients' reported benefit from previous treatment. Data were available for 32/33 PwMS and 52/55 PSS subjects; perceived efficacy rates were similar between the two groups (Table 2). We compared the clinical characteristics of subjects who reported benefit from treatment (*n* = 74) with those who did not experience improvement after BoNT-A injections (*n* = 10). Subjects who reported benefit underwent a higher number of treatments and were more commonly treated with incobotulinumtoxinA (Supplementary Table S3). A binary logistic regression showed that the overall model was significant, *χ*^2^(2) = 15.59, *p* < 0.001, with a Nagelkerke *R*^2^ of 0.327. The model indicated that the use of incobotulinumtoxinA was a significant predictor of a positive treatment response (*B* = 2.244, *p* = 0.041). The number of treatments showed a positive but non-significant association with the outcome (*B* = 0.522, *p* = 0.052; OR = 1.69, 95% CI: 0.996–2.85).

## 4 Discussion

This study primarily aimed to describe spasticity management with BoNT-A injections in 33 PwMS and 55 PSS patients in the real-life setting of a single-center Spasticity Clinic.

### 4.1 Patients’ characteristics and spasticity pattern

As expected, PwMS were younger, in line with MS and stroke epidemiology; MS typically has a peak incidence at 30–34 years (32), whereas more than 90% of strokes occur after 50 years of age (33). Moreover, PwMS showed a longer disease duration compared with PSS patients. This aligns with previous evidence, as spasticity is more common in advanced MS phases (34), while in more than 50% of post-stroke patients it develops within 6 months after the cerebrovascular event (35). Accordingly, in our cohort PwMS exhibited high levels of disability, with a median EDSS of 7 and a predominantly progressive disease course, and a consequently low proportion of patients treated with DMDs (∼30%).

Distinct spasticity phenotypes were observed between the two conditions. PwMS exhibited higher rates of paraspasticity and tetraspasticity, possibly related to frequent involvement of spinal cord pathways in MS, whereas PSS patients showed a more focal and lateralized distribution, with significantly higher frequencies of monolateral upper-limb spasticity and hemispasticity. These observations are consistent with previous literature describing more bilateral and diffuse spasticity patterns in MS (36), and more focal or hemibody distributions after stroke (37).

Within the PSS group, no significant differences were observed between ischemic and hemorrhagic stroke patients in demographic, clinical features, or treatment-related variables. Although patients with hemorrhagic stroke are reported to have a higher risk of developing spasticity compared with those with ischemic stroke (35, 38), no consistent differences in spasticity patterns between these two subtypes have been described. This suggests that, once spasticity has developed, the underlying stroke subtype may have limited influence on chronic PSS patterns and treatment strategies, as reflected by the absence of different treatment guidelines for the two conditions (8).

### 4.2 Treated limbs and BoNT-A dose

In our study, upper limbs were rarely treated in MS (*n* = 2, 6.1% of cases), whereas lower limbs were treated in almost 85% of PwMS and in fewer than 55% of patients with PSS. Patients with PSS were more frequently treated in the upper limbs. PwMS were more frequently treated with incobotulinumtoxinA. Moreover, in our experience, fewer than 60% of PwMS were treated in all affected limbs compared with almost all PSS subjects.

Both incobotulinumtoxinA and onabotulinumtoxinA dosages were lower in PwMS compared with patients with PSS, as confirmed in the multivariate analysis. No significant differences emerged when upper and lower limbs were analyzed separately, probably due to the small sample size. To evaluate the role of baseline disability as a determinant of BoNT-A dose, we conducted stratified analyses according to disease, given the different disability scales used (EDSS and NIHSS). In PwMS group, EDSS was the only significant variable associated with BoNT-A dose, while no significant factors were identified for PSS. The first study (25) that performed a similar comparison reported higher BoNT-A doses injected into the lower limbs of PwMS compared with patients with stroke. The authors suggested that this difference could be explained by variations in the pathogenesis and severity of spasticity, residual motor function, size, and pattern of injected muscles. Unfortunately, we did not perform a specific analysis of residual motor function or spasticity severity; therefore, it is not possible to fully evaluate all determinants of BoNT-A dosage. Nevertheless, the lower percentage of PwMS treated in all limbs affected by spasticity reflect the clinician's attempt to preserve residual function by avoiding excessive BoNT-A dosing. This interpretation is also supported by the positive association between total BoNT-A dose and higher EDSS, since in patients with greater disability preserving lower-limb motor function becomes less relevant, while other therapeutic goals—such as facilitating nursing care and hygiene—become more prominent.

Beyond these clinical drivers, pathophysiology may also play a role. In particular, spinal spasticity (more frequently observed in MS) is associated with positive phenomena such as spasms and clonus, which may require disease-specific BoNT-A treatment strategies (19, 39). Taken together, these factors may have contributed to the observed differences between groups. On the other hand, a more recent multicenter study showed that higher doses of abobotulinumtoxinA were required in the PSS group compared with MS, confirming our findings (28).

### 4.3 Treated muscles

Regarding injection patterns, we found that, unlike PSS, upper-limb involvement is rare in MS, in agreement with previous literature. Indeed, only about 7% of PwMS present upper-limb spasticity, with the “adducted shoulder” pattern being the most common (40). In our experience, treating upper-limb spasticity in MS, particularly in distal muscles, may be challenging due to the frequent absence of marked hypertonia and the presence of limited residual function, which we believe could be further compromised by BoNT-A treatment. In contrast, upper-limb involvement represents a major clinical issue in patients with PSS (28, 41), and in our cohort, wrist and finger flexors were predominantly affected. The pectoralis major was the only muscle commonly injected in both MS and PSS, as it contributes to the “pathological adducted shoulder” pattern in both conditions (42, 43).

With respect to lower-limb spasticity, PwMS were more frequently injected in the adductor muscles and rectus femoris, which are mainly involved in the “adducted thighs” and “stiff knee” spasticity patterns, respectively (22, 25, 27). Patients with PSS were more frequently injected in the soleus and tibialis posterior muscles, which are involved in the “equinovarus foot” pattern (44, 45). Previous reports have shown that hamstrings and hip adductors are the most frequently injected muscles in MS, whereas toe flexors are more commonly treated in PSS (24, 28).

### 4.4 Treatment efficacy

Perceived treatment efficacy was comparable between groups. Butera et al. also reported that the vast majority of subjects were satisfied with the treatment (28). Nevertheless, while in their study higher satisfaction ratings were observed in the PSS group, in our population perceived treatment efficacy was similar between groups. This difference could be explained by their more refined assessment tool, which used a 4-level Global Treatment Satisfaction Rating, whereas we were only able to collect a dichotomous evaluation of effect (28).

In our cohort, patients reporting clinical benefit had undergone a higher number of treatment sessions and were more frequently treated with incobotulinumtoxinA. Logistic regression analysis confirmed that incobotulinumtoxinA use was significantly associated with higher odds of perceived benefit, while the number of infiltrations showed a positive but borderline association.

To our knowledge, no studies have directly compared the clinical efficacy of incobotulinumtoxinA and onabotulinumtoxinA in MS- or stroke-related spasticity. The two toxins have been compared for the treatment of glabellar frown lines, showing similar efficacy one month after treatment when the same number of units was injected (46). Similar findings were reported in a crossover study on cervical dystonia (47) where a 1:1 conversion ratio was also used. However, literature focused on blepharospasm suggests that a 1:1 dose ratio may not produce equivalent outcomes (48), with incobotulinumtoxinA potentially requiring higher doses (proposed incobotulinumtoxinA: onabotulinumtoxinA conversion ratio of 1.2:1) (49) and shorter injection intervals, as confirmed by a real-world study in patients with blepharospasm or cervical dystonia switching from onabotulinumtoxinA to incobotulinumtoxinA for administrative or financial reasons (30). In our opinion, our study does not allow us to draw definitive conclusions regarding the potential influence of BoNT-A formulation on real-world treatment response, due to the lack of standardized outcome measures, such as validated spasticity scales (e.g., Modified Ashworth Scale, Tardieu Scale), spasm scales (e.g., Penn Spasm Frequency Scale), pain measures (e.g., NRS), or Goal Attainment Scaling.

### 4.5 Study limitations

The main limitations of our study include its retrospective design, which resulted in the lack of data regarding standardized spasticity, spasm, and pain scales, as well as treatment goals and functional outcomes. These elements could have further clarified the determinants of BoNT-A dosage. Moreover, the sample size was relatively small; consequently, the results of the predictive models should be interpreted with caution. Finally, as a monocentric study, our observations may reflect local practice patterns rather than underlying pathophysiological differences. Despite these limitations, our study provides interesting insights into BoNT-A–based treatment approaches for PSS and MS-related spasticity, reflecting our clinical practice aimed at optimizing spasticity control while preserving residual motor function.

## 5 Conclusions

This study shows differences in the clinical presentation and management of spasticity with BoNT-A between PwMS and patients with PSS. PwMS more frequently exhibit bilateral, lower-limb-dominant spasticity, whereas post-stroke patients typically present with more focal, unilateral patterns, particularly affecting the upper limbs. These differences are reflected in treatment strategies, as PwMS were more often treated in the lower limbs, while patients with PSS were more frequently treated in the upper limbs. PwMS also received lower overall doses of both onabotulinumtoxinA and incobotulinumtoxinA compared with PSS patients. Despite variations in injection patterns and dosing, perceived treatment benefit was similar across groups, supporting the effectiveness of BoNT-A therapy when individualized according to clinical characteristics and patient needs.

## Data Availability

The raw data supporting the conclusions of this article will be made available by the authors, without undue reservation.
