# Supplementary material for: Botulinum toxin treatment for multiple sclerosis and post-stroke spasticity in clinical practice: differences in injection doses and patterns
Source: Front Rehabil Sci. 2026 Jun 9;7:1838788. doi: 10.3389/fresc.2026.1838788 (PMC13286915; doi:10.3389/fresc.2026.1838788)
Supplement: Supplementary file 1 [file Datasheet1.pdf]

Supplementary Material

1 Supplementary Tables

Table S1. Demographic and clinical data in post-stroke spasticity

	Ischemic stroke (n = 44)	Hemorrhagic stroke (n = 11)	p
**Age**	71 (39 - 86)	68 (54 - 79)	0.600[a]
**Sex (m)**	34 (77.3%)	7 (63.6%)	0.443[b]
**Treatment duration (years)**	0,9 (0 - 4)	0,5 (0 – 3.5)	0.270[a]
**Number of treatments**	3 (1 - 10)	3 (1 - 11)	0.848[a]
**Disease duration (years)**	3 (0 - 18)	3 (1 -11)	0.850[a]
**NIHSS**	6 (3 - 12)	7 (3 - 14)	0.273[a]
**BoNT-A total dose (U)**			
**IncobotulinumtoxinA**	225 (25 - 400)	200 (100 - 400)	0.535[a]
**OnabotulinumtoxinA**	212.5 (25 - 400)	150 (100 - 300)	0.599
**Upper limb injections**	34 (77.3%)	9 (81.8%)	1.000[b]
**IncobotulinumtoxinA, N, dose (U)**	N=10, 140 (50-300)	N=4, 200 (80 - 400)	0.474[a]
**OnabotulinumtoxinA, N, dose (U)**	N=23, 200 (50-400)	N=5, 100 (100 - 300)	0.244[a]
**Lower limb injections**	25 (56.8%)	5 (45.5%)	0.498[b]
**IncobotulinumtoxinA, N, dose (U)**	N=7, 190 (50-200)	N=1, 50	0.174[a]
**OnabotulinumtoxinA, N, dose (U)**	N=17, 150 (25-300)	N=4, 150 (100-300)	0.647[a]
**Ipsilateral upper and lower limb injections**	15 (34.1%)	3 (27.3%)	1.000[b]
**Patients' reported efficacy**	34 (82.9%)	11 (100%)	0.322[b]

Expressed as "median (range)" or "number (percentage)". [a] Mann-Whitney U test; [b] Fisher's exact test. BoNT-A: Botulinum toxin-A.

Table S2. Muscles treated with botulinum toxin-A and botulinum toxin-A dosage*.

	MS group (n=33)	PSS group (n=55)	p	MS group (n=33)	PSS group (n=55)	p
	Muscle injected (n %)			BoNT-A dosage (U)*		
**Upper limb**						
Bicepes brachii	3 (9.1%)	18 (32.7%)	0.012[a]	65 (50 - 75)	55 (30 - 150)	0.752[b]
Brachioradialis	2 (6.1%)	9 (16.4%)	0.198[c]	50 (50 - 50)	50 (10 - 150)	0.797[b]
Brachial	1 (3%)	0 (0%)	0.375[c]	75		
Pronator teres	0 (0%)	7 (12.7%)	0.042[c]		50 (50 - 70)	
Palmar	0 (0%)	1 (1.8%)	1.000[a]		30	
Flexor carpi radialis	0 (0%)	14 (25.5%)	0.002[a]**		50 (25 - 80)	
Flexor carpi ulnaris	0 (0%)	16 (29.1%)	<0.001[a]**		50 (25 - 100)	
Flexor digitorum superficialis	0 (0%)	24 (43.6%)	<0.001[a]**		50 (25 - 75)	
Flexor digitorum profundus	0 (0%)	19 (34.5%)	<0.001[a]**		50 (25 - 200)	
Flexor pollicis longus	0 (0%)	2 (3.6%)	0.526[c]		50 (50 - 50)	
Opponens pollicis	0 (0%)	1 (1.8%)	1.000[c]		30	
Interosseus muscles	0 (0%)	17 (30.9%)	<0.001[a]**		40 (20 - 100)	
Pectoralis major	4 (12.1%)	8 (14.5%)	1.000[c]	75 (60 - 100)	50 (30 - 100)	0.191[b]
Triceps brachialis	1 (3%)	9 (16.4%)	0.083[c]	65	100 (25 - 200)	0.856[b]
Subscapularis	1 (3%)	0 (0%)	0.375[c]	20		
Deltoid	0 (0%)	1 (1.8%)	1.000[c]		50	
**Lower limb**						
Flexor hallucis longus	1 (3%)	0 (0%)	0.375[c]	30		
Flexor digitorum longus	1 (3%)	1 (1.8%)	1.000[c]	15	50	
Extensor hallucis longus	0 (0%)	2 (3.6%)	0.526[c]		100 (50 -100)	
Adductor muscles	7 (21.2%)	2 (3.6%)	0.013[c]	100 (50 - 100)	50 (50 - 50)	0.119[b]
Gastrocnemius	4 (12.1%)	8 (14.5%)	1.000[c]	37.50 (10 - 100)	60 (30 - 100)	0.218[b]
Hamstring muscles	6 (18.2%)	6 (10.9%)	0.354[c]	87.50 (50 - 150)	60 (50 - 100)	0.315[b]
Tibialis posterior	3 (9.1%)	19 (34.5%)	0.008[a]**	50 (20 - 50)	100 (25 - 150)	0.078[b]
Rectus femori	10 (30.3%)	3 (5.5%)	0.003[c]**	40 (20 - 150)	50 (20 - 70)	0.864[b]
Soleus	11 (33.3%)	24 (43.6%)	0.339[a]	50 (25 - 100)	70 (25 - 100)	0.019[b]

Expressed as "median (range)" or "number (percentage)". [a] Chi square test; [b] Mann-Whitney U test; [c] Fisher's exact test. MS: multiple sclerosis; PSS: post-stroke spasticity.
*1:1 conversion ratio between incobotulinumtoxinA and onabotulinumtoxinA, despite not universally accepted, has been applied to allow comparison between muscle groups.
** Statistical significance confirmed, after Benjamini-Hochberg procedure was applied to control the false discovery rate (FDR).

Table S3. Comparison between patients reporting benefit and those reporting no benefit from botulinum toxin-A treatment.

	BoNT-A benefit (n = 74)	No BoNT-A benefit (n = 10)	p
**Diagnosis**			
**MS**	29 (90.6%)	3 (9.4%)	0.735[a]
**PSS**	45 (86.5%)	7 (13.5%)	
**Age (years)**	65.5 (24 -86)	70.5 (42 - 77)	0.275[b]
**Sex**			
**f**	25 (92.6%)	2 (7.4%)	0.489[a]
**m**	49 (86%)	8 (14%)	
**Disease duration (years)**	6 (0 - 48)	3.5 (2 - 21)	0.835[b]
**Treatment duration (years)**	0 (0 - 4)	0 (0 - 1)	0.077[b]
**Number of treatments**	3 (1 - 12)	2 (1 - 3)	0.009[b]
**Type of spasticity**			0.298[c]
**Upper limb, monolateral**	21 (28.4%)	3 (30.0%)	
**Upper limb, bilateral**	1 (1.4%)	0 (0%)	
**Lower limb, monolateral**	16 (21.6%)	0 (0%)	
**Lower limb, bilateral (paraspasticity)**	10 (13.5%)	3 (30.0%)	
**Hemispasticity**	17 (23%)	4 (40.0%)	
**Tetraspasticity**	9 (12.2%)	0 (0%)	
**NIHSS**	6 (3-14)	7 (6-11)	0.118[b]
**EDSS**	7 (2 – 8.5)	6.5 (5-6.5)	0.439[b]
**MS disease form**			0.727[c]
**Relapsing-remitting**	7 (24.1%)	1 (33.3%)	
**Secondary progressive**	17 (58.6%)	2 (66.7%)	
**Primary progressive**	5 (17.2%)	0 (0%)	
**Toxin injected (n, %)**			
**IncobotulinumtoxinA**	38 (97.4%)	1 (2.6%)	0.017[a]
**OnabotulinumtoxinA**	36 (80.0%)	9 (20.0%)	
**BoNT-A total dose (U)**			
**IncobotulinumtoxinA**	200 (20-400)	150	0.559[b]
**OnabotulinumtoxinA**	200 (25-400)	200 (50-325)	0.458[b]
**Number of limbs injected**	1 (1 - 2)	1.5 (1 - 2)	0.629[b]
**Number of muscles injected**	3 (1 - 8)	2.5 (1 - 6)	0.983[b]
**Patients treated in upper limb(s), (n, %)**	40 (54.1%)	7 (70.0%)	0.501[a]
**IncobotulinumtoxinA dose (U) max**	200 (50-400)	N=1, 150	0.875[b]
**OnabotulinumtoxinA dose (U) max**	N= 24, 200 (70-400)	N= 6, 137.5 (50-325)	0.273[b]
**bilateral injections**	2 (2.4%)	0 (0%)	1.000[a]
**number of muscles injected**	1 (0 - 7)	2 (0 - 5)	0.567[b]
**Patients treated in lower limb(s), (n, %)**	49 (66.2%)	6 (60.0%)	0.731[a]
**IncobotulinumtoxinA dose (U) max**	N=29, 100 (20-250)	N=0, 0	NA
**OnabotulinumtoxinA dose (U) max**	N= 19, 100 (25-300)	N=6, 112.5 (50-200)	0.366[b]
**bilateral injections**	14 (18.9%)	1 (10.0%)	0.682[a]
**number of muscles injected**	1 (0 - 4)	1 (0 - 2)	0.603[b]
**Ipsilateral upper and lower limb injections**	15 (20.3%)	3 (30%)	0.440[a]

[a] Fisher's exact test; [b] Mann-Whitney U test; [c] Pearson's chi-square. BoNT-A: Botulinum toxin-A. MS: multiple sclerosis; PSS: post-stroke spasticity; NIHSS: National Institutes of Health Stroke Scale; EDSS: Expanded Disability Status Scale.

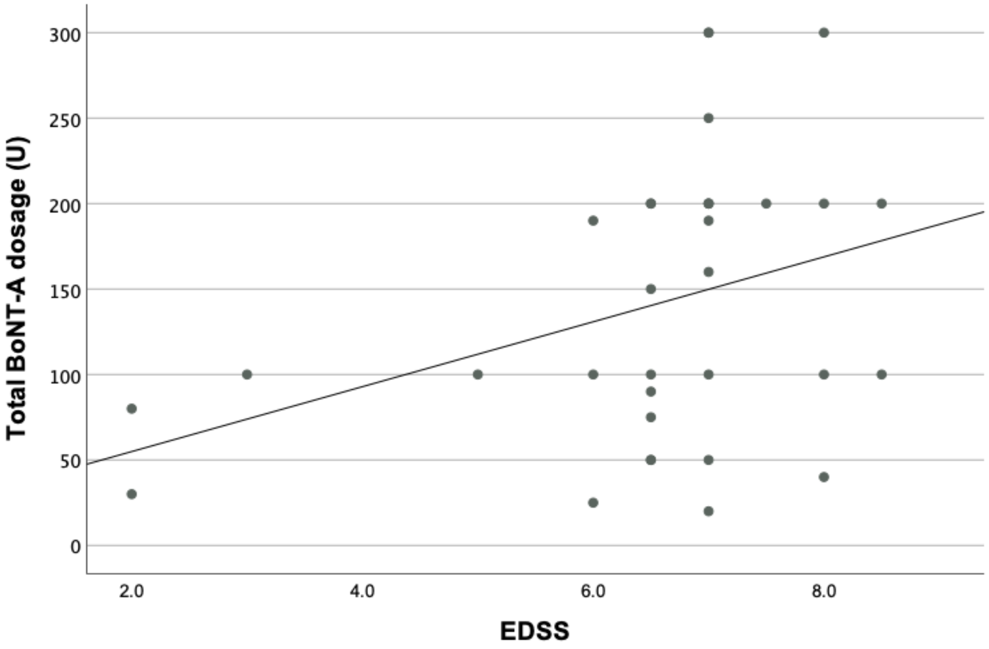

Figure S1. Positive correlation between total Botulinum Toxin Type A (BoNT-A) dosage and disability (EDSS) in multiple sclerosis group (Spearman's rho=0.399, p=0.021).
